# Multimeric Amphipathic α‐Helical Sequences for Rapid and Efficient Intracellular Protein Transport at Nanomolar Concentrations

**DOI:** 10.1002/advs.201800240

**Published:** 2018-06-19

**Authors:** Jae Hoon Oh, Seung‐Eun Chong, Sohee Nam, Soonsil Hyun, Sejong Choi, Hyojun Gye, Sangmok Jang, Joomyung Jang, Sung Won Hwang, Jaehoon Yu, Yan Lee

**Affiliations:** ^1^ Department of Chemistry Seoul National University 1 Gwanak‐ro Gwanak‐gu Seoul 08826 Republic of Korea; ^2^ Department of Chemistry and Education Seoul National University 1 Gwanak‐ro Gwanak‐gu Seoul 08826 Republic of Korea; ^3^ Department of Nano Science and Mechatronics Engineering Konkuk University 268 Chung Won Dae Ro Chungju‐City Chungbuk 380‐701 Republic of Korea

**Keywords:** adipocyte differentiation, cell penetrating peptides, intracellular protein transport, multimeric amphipathic α‐helical peptides, nanomolar concentrations

## Abstract

An amphipathic leucine (L) and lysine (K)‐rich α‐helical peptide is multimerized based on helix‐loop‐helix structures to maximize the penetrating activities. The multimeric LK‐based cell penetrating peptides (LK‐CPPs) can penetrate cells as protein‐fused forms at 100–1000‐fold lower concentrations than Tat peptide. The enhanced penetrating activity is increased through multimerization by degrees up to the tetramer level. The multimeric LK‐CPPs show rapid cell penetration through macropinocytosis at low nanomolar concentrations, unlike the monomeric LK, which have slower penetrating kinetics at much higher concentrations. The heparan sulfate proteoglycan (HSPG) receptors are highly involved in the rapid internalization of multimeric LK‐CPPs. As a proof of concept of biomedical applications, an adipogenic transcription factor, peroxisome proliferator‐activated receptor gamma 2 (PPAR‐γ 2), is delivered into preadipocytes, and highly enhanced expression of adipogenic genes at nanomolar concentrations is induced. The multimeric CPPs can be a useful platform for the intracellular delivery of bio‐macromolecular reagents that have difficulty with penetration in order to control biological reactions in cells at feasible concentrations for biomedical purposes.

## 1 Introduction

Bio‐macromolecules such as DNA, RNA, and proteins are considered to be potential biomedical reagents for the control of selective biological reactions.^1^ Since most bio‐macromolecules have difficulty penetrating cells through their plasma membranes, various methodologies based on viral and nonviral delivery systems have been applied for the intracellular delivery of bio‐macromolecules.^2^ Although viral systems show outstanding delivery efficiency, they often face critical safety issues in clinical trials.[[qv: 2a,3]] On the other hand, nonviral systems exhibit rather limited delivery efficiency, but are relatively free from safety issues.[[qv: 2a,4]] Thousands of chemical and biological strategies have been challenged to overcome the biocompatibility and delivery efficiency barriers of conventional techniques.^5^

Existing between the chemical and biological strategies, cell penetrating peptides (CPPs) and protein transduction domains (PTDs) are highlighted as powerful tools for facilitating the intracellular delivery of bio‐macromolecules.^6^ After the first discovery of the Tat sequence from a human immunodeficiency virus (HIV) protein,^7^ a number of CPPs have been applied to the intracellular translocation of hard‐to‐penetrate biomolecules.^8^ Chemically synthesized CPPs are covalently or noncovalently hybridized to biomedical reagents to enhance cell penetration and drug efficacy.^9^ Moreover, CPPs are highly desirable for intracellular protein delivery because CPP sequences can be readily inserted into protein sequences using genetic recombination techniques.^10^ Various proteins have been delivered into the cell interiors in CPP‐fused forms to correct disordered biochemical reactions,^11^ induce apoptosis,^12^ or control cell lineages^13^ for corresponding biomedical objectives.

CPPs have many advantages over other delivery techniques. They are capable of simple and systematic synthesis by both chemical and biological methods, high biocompatibility due to naturally originated amino acid–based structures, efficient tissue penetration, and may be combined in a versatile fashion with both viral and nonviral delivery systems. However, one of the greatest weaknesses impeding the broad biomedical application of CPPs is the penetration concentration, i.e., the extracellular concentration threshold showing significant penetration of peptides. Most CPPs or CPP‐conjugated cargos are internalized into cells only at micromolar concentrations.^14^ The micromolar penetrating concentrations may be acceptable for the delivery of small molecules, but it is expensive and impractical to administer micromolar doses of bio‐macromolecules. We believe that it is essential to discover or develop new CPPs that are able to deliver macromolecular cargo into cells at clinically feasible concentrations for future practical CPP‐based therapeutic applications.

The CPPs that have previously been reported are generally categorized into two structural types, multi‐positively charged sequences and amphipathic cationic sequences. In this study, we found that the multimerization of amphipathic α‐helical peptide sequences could greatly reduce the penetrating concentration and accelerate the penetration rate of the CPPs. Particularly in case of over tetrameric sequences, the multimeric peptides showed rapid protein internalization within 30 min through direct penetration or unique endocytic mechanisms at nanomolar concentrations. Shorter sequences or conventional CPPs showed a much slower internalization through different endocytic mechanisms even at far higher micromolar concentrations. As the first step of biomedical application, we were able to induce adipocyte differentiation effectively by delivering the multimeric CPP‐fused form (tetrameric form) of a key transcription factor called peroxisome proliferator‐activated receptor gamma 2 (PPAR‐γ  2) at nanomolar concentrations.

## 2 Results and Discussion

### 2.1 Nanomolar Cell Penetration of LK Multimer

We have recently discovered that the penetrating concentration of a 16‐meric amphipathic α‐helical peptide (LKKLCKLLKKLCKLAG; leucine (L) and lysine (K)‐rich α‐helical (LK) peptide) could be greatly reduced by dimerization between two LK‐peptide molecules through the oxidative formation of two disulfide bonds.^15^ Remarkably, the dimeric peptide with an antiparallel structure as the major form can penetrate cells and inhibit the RNA transcription of HIV genes at nanomolar concentrations over 100‐fold lower than to the penetrating concentrations of other conventional CPPs such as Tat or oligoarginines.

**Figure 1:**
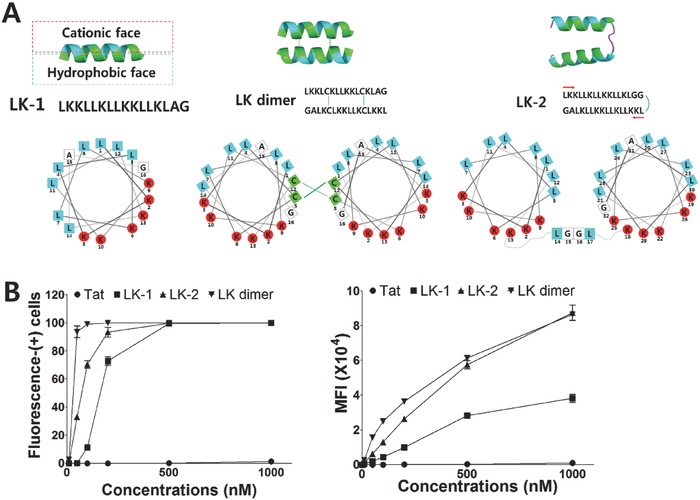
LK peptide and derivatives cell penetrating efficiency. A) Simplified sequences and structural representation of LK monomer (LK‐1), LK dimer with two disulfide bonds, and LK‐2. The structure of each peptide was predicted by PEP‐FOLD (http://bioserv.rpbs.univ-paris-diderot.fr/services/PEP-FOLD/). B) Cell penetrating activities of TAMRA‐labeled peptides on HeLa cells after 12 h of incubation. Fluorescence‐(+) cells were analyzed by FACS. MFI means mean fluorescence intensity of cells in FACS data. Data points are represented as the average value of three experiments ± standard deviation.

In nature, α‐helical bundles such as helix‐loop‐helix (HLH) and helix‐turn‐helix motifs are frequently observed in coiled coil proteins.^16^ The antiparallel LK dimer has a close resemblance to that of natural HLH motifs with the exception of the fact that the monomeric units in the dimer are linked via disulfide bonds. We expected that an HLH motif with a similar sequence could mimic the antiparallel LK dimer for high cell penetrating activity. The HLH motif can be linearly synthesized through chemical methods such as solid‐phase peptide synthesis (SPPS) with higher yields than the disulfide‐based LK dimer which requires an additional dimerization step. Furthermore, the HLH motif can also be prepared and readily fused with cargo proteins through genetic recombination. Therefore, starting from LKKLLKLLKKLLKLAG (LK‐1), which replaced the cysteines (C) of the LK peptide with leucines (L) for the prevention of premature and irregular disulfide formation during protein translation, we prepared LK‐2 with a linearly duplicated sequence of the LK‐1 and glycine–glycine (GG) dipeptides in the middle of the sequence (**Figure** **1**A). We intended for the GG sequence to be able to form a flexible turn (or loop) between two α‐helices for the antiparallel HLH structure.^17^ The α‐helical propensity of LK‐2 was significantly enhanced in comparison to LK‐1 as shown in the circular dichroism (CD) spectra (Figure S1, Supporting Information). Especially, LK‐2 showed an α‐helical content of ≈85% in a membrane‐mimic condition. The α‐helical structure of LK‐1 could be stabilized by the linear duplication of the sequences probably due to the helix–helix interactions.

The cell penetrating activity of LK‐2 was evaluated in a human cervical cancer cell line (HeLa) through fluorescence‐activated cell sorting (FACS). The disulfide‐based LK‐dimer and Tat sequence (11 residues, YGRKKRRQRRR) were used as references. The cell penetration activities of the fluorescence‐labeled peptides were found to increase in the following order: Tat < LK‐1 < LK‐2 < LK‐dimer (Figure 1B). The LK‐2 displayed slightly lower cell penetrating activity than the LK‐dimer; however, there was >30% penetration at 50 × 10^−9^ m, and nearly quantitative penetration at 200 × 10^−9^ m. On the other hand, Tat showed only 1% cell penetration even at 1 × 10^−6^ m. Clearly, the linear duplicate of the LK peptide that mimics the LK‐dimer has much higher cell penetrating activity than conventional CPPs.

**Figure 2:**
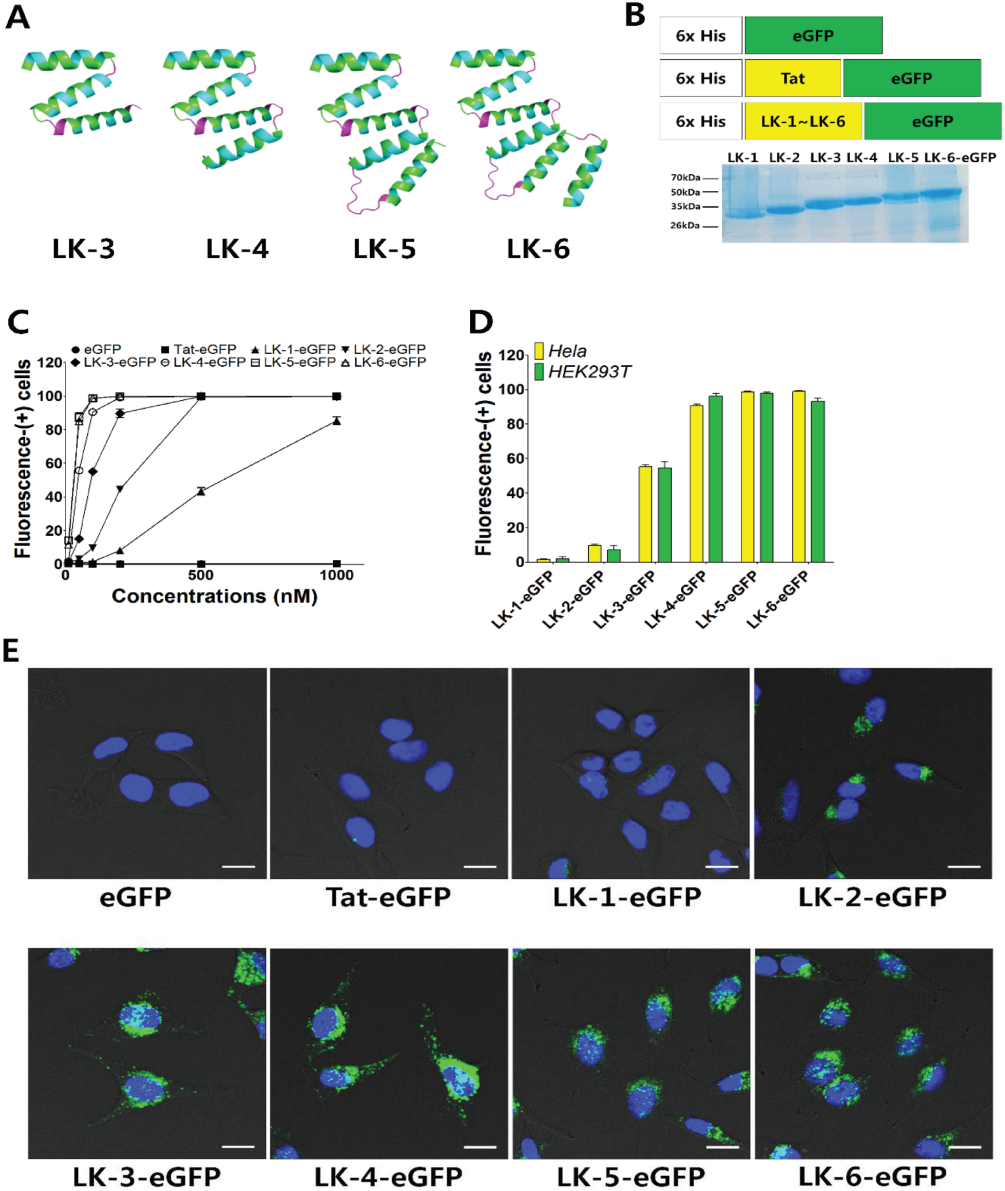
Cell penetrating activity of LK‐fused eGFP. A) Schematic representation of LK‐3, LK‐4, LK‐5, and LK‐6 structures. The helix and HLH structure were predicted by PEP‐FOLD. B) Construction of CPP‐fused eGFP and the sodium dodecyl sulfate‐polyacrylamide gel electrophoresis (SDS‐PAGE) results of purified proteins. C) Cell penetration activities of CPP‐fused eGFPs on HeLa cells after 12 h of incubation. Fluorescence‐(+) cells were analyzed by FACS. D) Comparison of fluorescence‐(+) cells on HeLa and HEK293T cells after 12 h of incubation with each CPP‐fused eGFP at 100 × 10^−9^ m. E) CLSM images of HeLa cells after 12 h of incubation with each CPP‐fused eGFP at 100 × 10^−9^ m. The intracellular localization of eGFPs was visualized as green and the Hoechst 33342‐stained nucleus was shown as blue. The scale bar represents 20 µm. All data points are represented as the average value of three experiments ± standard deviation.

We further investigated the effect of the multimerization of the LK sequence. Notations LK‐3, LK‐4, LK‐5, and LK‐6 were used for the trimeric, tetrameric, pentameric, and hexameric sequences (Table S1, Supporting Information) of the LK‐1 peptide, respectively, as represented in **Figure** **2**A. Since it is difficult to synthesize peptides with over 50 amino acids through chemical methods, fused proteins with each sequence at the N‐terminus of a cargo protein were instead prepared by genetic recombination. The linker peptide sequences between the LK monomers in multimeric sequences were somewhat varied due to limit of recombinable sequences in the plasmid where highly repeated sequences could be inserted in the genes for the LK multimers. Enhanced green fluorescence protein (eGFP) was used as a model protein to compare the cell penetrating activities of the multimeric sequences through the measurement of fluorescence. CPP‐fusion proteins, including Tat–eGFP as a positive control, were successfully prepared by plasmid transformation into *Escherichia coli* and purified by affinity chromatography based on N‐terminal His‐tags (Figure 2B).

The cell penetrating activities of CPP‐fused eGFPs were compared in two cell lines using sub‐micromolar concentrations, at which Tat and oligoarginine showed only limited cell penetration. Figure 2C and Figure S2 (Supporting Information) showed the concentration‐dependent penetration efficiency on the HeLa (human cervical cancer) and HEK 293T (human embryonic kidney) cells after a 12 h of treatment using CPP‐fused eGFPs. The cell penetrating activities of the CPP‐fused eGFPs were dramatically increased by the multimerization of the LK sequences on both cell lines. The Tat–eGFP showed almost negligible penetrating efficiency below 1 × 10^−6^ m, but the LK‐1–eGFP showed penetrating activity with 40% and 80% of cell being fluorescence‐positive (+) at 500 × 10^−9^ m and 1 × 10^−6^ m, respectively. The LK‐2–eGFP with a dimerized CPP showed 40% at 200 × 10^−9^ m and almost 100% at 500 × 10^−9^ m. The LK‐3–eGFP showed even higher cell penetrating activity with over 50% at 100 × 10^−9^ m. The enhancement of cell penetrating activity through multimerization was almost saturated over tetramerization. LK‐4–, LK‐5–, and LK‐6–eGFP all showed over 50% and almost 100% of cells being fluorescence‐(+) at 50 × 10^−9^ m and 100 × 10^−9^ m, respectively. Figure 2D compares the cell penetrating activities of CPP‐fused eGFPs at 100 × 10^−9^ m, and illustrates the sharp increase of cell penetrating activities at LK‐3–eGFP and saturation after LK‐4–eGFP. The intracellular green fluorescence of CPP–eGFPs was visualized at 100 × 10^−9^ m concentration through confocal laser scanning microscopy (CLSM) (Figure 2E). Corresponding to the FACS data, the numbers and intensities of green pixels clearly increased from LK‐1–eGFP to LK‐6–eGFP. Under the same conditions, only negligible green fluorescence was observed in Tat–eGFP‐treated cells. Most CPP–eGFPs were observed in the cytoplasm although it is still unclear whether the internalized proteins escaped from endosome or not. However, a small portion of them were localized in the nuclei stained with 4′,6‐diamidino‐2‐phenylindole (DAPI), representing co‐localized cyan signals from green and blue fluorescence. The multimeric CPP–eGFPs showed almost no cytotoxicity up to 1 µm, which was far over the effective penetrating concentration of 100 × 10^−9^ m (Figure S3, Supporting Information).

### 2.2 The Cell Penetrating Mechanism of LK Multimers

**Figure 3:**
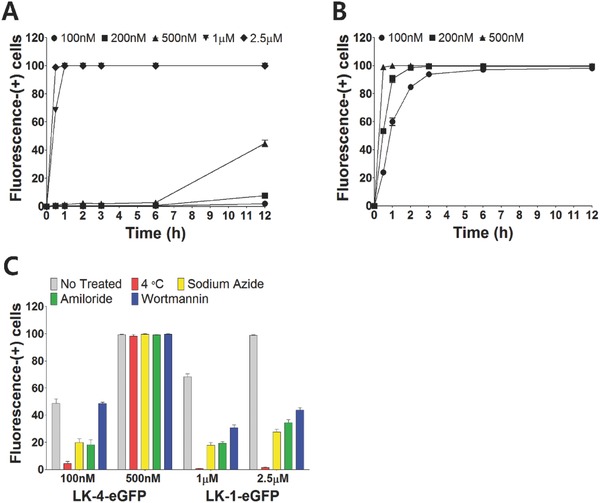
Cell penetrating kinetics and mechanism of LK multimers. Cell penetrating kinetics of A) LK‐1–eGFP and B) LK‐4–eGFP on HeLa cells at various concentrations. C) Inhibition of penetration of LK‐1–eGFP and LK‐4–eGFP into HeLa cells. Cells were pretreated with various inhibitory conditions for 1 h (4 °C) or 3 h (others), and further incubated with LK–eGFPs at 37 °C for 1 h. All data points are represented as the average value of three experiments ± standard deviation.

We examined the outstanding cell penetrating properties of the multimeric CPPs in more detail by comparing the penetrating kinetics of LK‐1–eGFP and LK‐4–eGFP at various concentrations. LK‐1–eGFP only began to penetrate into HeLa cells after 6 h of incubation at concentrations below 500 × 10^−9^ m (**Figure** **3**A). However, at 1 × 10^−6^ and 2.5 × 10^−6^ m, the penetration rate of LK‐1–eGFP accelerated, and 70% and 98% of cells were fluorescence‐(+) after a 30 min of incubation, respectively. On the other hand, the LK‐4–eGFP condition had over 60% of cells fluorescence‐(+) within 1 h at 100 × 10^−9^ m, and the rate increased with the elevation of the concentration (Figure 3B). At 200 × 10^−9^ and 500 × 10^−9^ m, LK‐4–eGFP penetrated 55% and 100% of cells within 30 min, respectively. On the basis of this result, we believe that the penetrating mechanism of LK‐based CPPs may be dependent upon both the multimerization degree and the concentration (Figure S4, Supporting Information).

CPPs are thought to be internalized into cells through both adenosine triphosphate (ATP)‐dependent and ATP‐independent mechanisms.^18^ Receptor‐mediated endocytosis such as clathrin‐ or caveolae‐mediated endocytosis and macropinocytosis requires ATP for the uptake of CPPs, whereas intracellular entry through direct penetration via fusion and aggregate formation on the membrane proceeds without ATP.^19^ Most CPPs penetrate cells using more than one mechanism.^20^ HeLa cells were pretreated with representative internalization inhibiting conditions: wortmannin (a receptor‐mediated endocytosis inhibitor), amiloride (a macropinocytosis inhibitor), sodium azide (NaN_3_, an ATP synthase inhibitor), and a 4 °C‐incubation (energy depletion),^21^ and then were further incubated with LK‐1–eGFP and LK‐4–eGFP at various concentrations for 1 h in order to compare the penetrating efficiency at the early stage. In the case of LK‐4–eGFP, the inhibition profile clearly changed according to the concentration (Figure 3C). At 100 × 10^−9^ m, LK‐4–eGFP showed ≈50% of cells being fluorescence‐(+) at the 1 h point. Energy depletion inhibited the penetration almost completely implying that the entry mechanism was ATP dependent. The significant inhibition of the penetrating activity through the sodium azide treatment also supported the concept that the internalization of LK‐4–eGFP was ATP dependent. By comparing the fluorescence in the wortmannin and amiloride treatments, which showed almost no inhibition and 70% inhibition, respectively, we concluded that LK‐4–eGFP predominantly utilizes ATP‐dependent endocytosis mechanisms at this low concentration, mainly macropinocytosis rather than receptor‐mediated endocytosis. The penetrating activity of LK‐4–eGFP at 500 × 10^−9^ m, however, was almost unaffected by the inhibition conditions. This result supported the thought that LK‐4–eGFP rapidly penetrated cells through ATP‐independent pathways at 500 × 10^−9^ m. A marginal increase in membrane destabilization was observed in the lactate dehydrogenase (LDH) assay at 500 × 10^−9^ m, but the destabilization degree was very limited (<6%; Figure S5, Supporting Information).

As shown in Figure 3A and 3B, the penetration of LK‐1–eGFP was much slower than LK‐4–eGFP at concentrations below 1 × 10^−6^ m, but was somewhat accelerated at the 1 × 10^−6^ and 2.5 × 10^−6^ m concentrations. Even in this high concentration, LK‐1–eGFP showed only ATP‐dependent penetration that was likely to be both receptor‐mediated endocytosis and macropinocytosis, based on the results of complete inhibition at 4 °C and the ≈50–70% inhibition in the sodium azide, wortmannin, and amiloride conditions.

**Figure 4:**
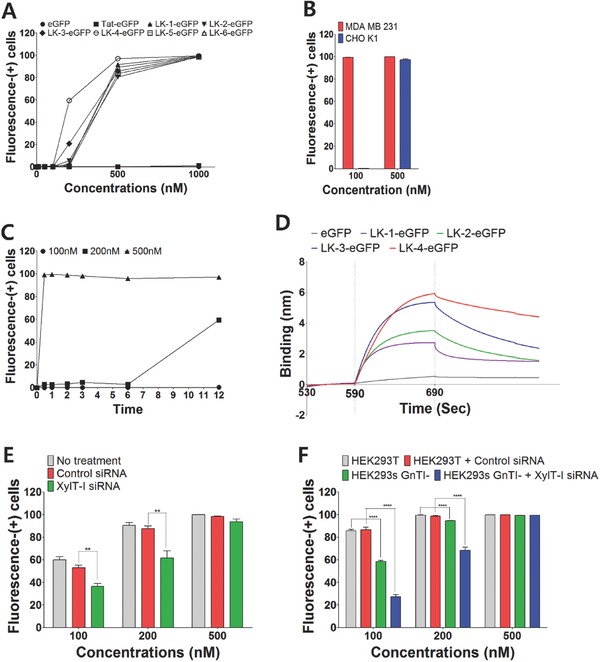
Effect of heparan sulfate on the entry of LK‐fused eGFPs. A) Cell penetration activities of CPP‐fused eGFPs on CHO‐K1 cells after 12 h of incubation. B) Comparison of fluorescence‐(+) cells on MDA‐MB‐231 and CHO‐K1 cells after 12 h of incubation with LK‐1–eGFP and LK‐4–eGFP at 100 × 10^−9^ m and 500 × 10^−9^ m. C) Cell penetrating kinetics of LK‐4–eGFP on CHO‐K1 cells at various concentrations. D) Comparison of the association and dissociation between LK‐fused proteins (2.5 × 10^−6^ m) and heparin which was measured by biolayer interferometry. eGFP (20 × 10^−6^ m) was used as a control. E) Comparison of fluorescence‐(+) cells on HeLa cells by small interfering RNA (siRNA)‐based inhibition of xylosyltransferase‐I (XylT‐I) expression. Cells were transfected by XylT‐I siRNA for 48 h and then treated with LK‐4–eGFP for 1 h. F) Comparison of fluorescence‐(+) cells on HEK 293 cell lines by siRNA‐based inhibition. HEK 293T and HEK293s GnTi^−^ cells were transfected by control siRNA and XylT‐I siRNA for 48 h and then treated with LK‐4–eGFP for 0.5 h. Fluorescence‐(+) cells were analyzed by FACS. All data points are represented as the average value of three experiments ± standard deviation. (**) and (***) indicate 0.001 ≤ *p* < 0.01 and 0.0001 ≤ *p* < 0.001, respectively.

It has often been reported that many cationic CPPs were internalized by the mediation of extracellular glycosaminoglycans such as heparan sulfate proteoglycans (HSPG).^22^ We wondered if HSPG would be the key mediator to induce the rapid internalization of multimeric CPPs at nanomolar concentrations. MDA‐MB‐231 (human breast cancer) cells, which expressed a massive amount of HSPG,^23^ and CHO‐K1 (Chinese hamster ovary) cells that lacked 3‐O‐sulfotransferase, the key enzyme for the HSPG maturation,^24^ were selected to examine the effect of HSPG on the penetration of LK–eGFPs. All types of LK–eGFPs showed increased penetration at nanomolar concentrations in MDA‐MB‐231 cells (Figure S6A,B, Supporting Information) compared to the results from HeLa and HEK 293T (Figure 2C; Figure S2, Supporting Information). However, LK–eGFPs showed very limited cellular penetration in CHO‐K1 cells in the concentration ranges up to 100 × 10^−9^ m and only began to penetrate cells over 200 × 10^−9^ m with low efficiency (**Figure** **4**A; Figure S6C, Supporting Information). Figure 4B compares the fluorescence‐(+) cells among MDA‐MB‐231 and CHO‐K1 treated with of LK‐4–eGFP at 100 × 10^−9^ and 500 × 10^−9^ m after 12 h of incubation. Interestingly, LK‐4–eGFP showed a large difference in penetrating activities (100% vs 0.5%) at 100 × 10^−9^ m, but the difference was almost negligible at 500 × 10^−9^ m, showing 100% and 97% on the HSPG‐rich MDA‐MB‐231 and the CHO‐K1 cells with immatured HSPG, respectively. In addition, the penetration of LK‐4–eGFP into CHO‐K1 cells only began after 6 h at 200 × 10^−9^ m (Figure 4C), unlike the rapid penetration into the HeLa cells, which occurred within 1 h (Figure 3B) at the same concentrations. On the other hand, the rapid internalization of LK‐4–eGFP was similarly observed at 500 × 10^−9^ m for both cell lines. Since the main entry mechanism of LK‐4–eGFP was ATP‐dependent endocytosis at 100 × 10^−9^ m but ATP‐independent penetration at 500 × 10^−9^ m, we hypothesized that HSPG may be an important mediator for rapid induction of endocytosis in multimeric CPPs internalization at low concentrations, but not for direct penetration at high concentrations.

In order to support this hypothesis, we measured the relative affinity of LK–eGFPs on heparin, which mimics heparan sulfate in HSPG, by using biolayer interferometry.^25^ The increase in the binding affinity in heparin was in accord with the increase of the degree of LK multimerization (Figure 4D). The enhanced binding of LK multimers may have the potential to rapidly induce ATP‐dependent endocytosis, mainly macropinocytosis. Interestingly, although the association rates of all LK–eGFPs did not show much difference from each other, the dissociation rates of LK‐2–eGFP and LK‐4–eGFP were significantly slower than LK‐1–eGFP and LK‐3–eGFP. It is thought that even‐numbered multimeric LK sequences have a tendency to bind to HSPG more effectively than odd‐numbered sequences through rather specific interactions that take place beyond the electrostatic interaction.

Furthermore, we inhibited the attachment of heparan sulfate chains to HSPG using a xylosyltransferase‐I (XylT‐I) siRNA treatment on HeLa cells.^26^ The rapid internalization of LK‐4–eGFP within 30 min at 100 × 10^−9^ m was reduced from 60% to 35% (Figure 4E). However, the inhibition was almost negligible at 500 × 10^−9^ m. In addition, we compared the penetrating activities of LK‐4–eGFPs on HEK 293T and HEK293s GnTI^−^ cells. HEK293s GnTI^−^ cells lack *N*‐acetylglucosaminyltransferase I (GnTI), another key enzyme for the elongation of complex polysaccharide chains on HSPG.^27^ The lack of GnTI clearly reduced the penetrating activity at 100 × 10^−9^ m (Figure 4F). Additional XylT‐I siRNA treatment on HEK293s GnTI^−^ cells further decreased the penetrating activity. Again, the inhibitory effect on penetration activities was lowered by increasing the concentrations, and almost negligible, at all, at 500 × 10^−9^ m. These results strongly support the hypothesis that the rapid internalization of multimeric CPPs is mainly dependent upon HSPG at low concentrations, but not significantly at high concentrations. Of course, there would be strong interactions between HSPG and multimeric CPPs at high concentrations, but the CPPs could also enter into cells through other mechanisms such as direct interaction with the lipid bilayer in an ATP‐independent manner at the high concentrations.

**Figure 5:**
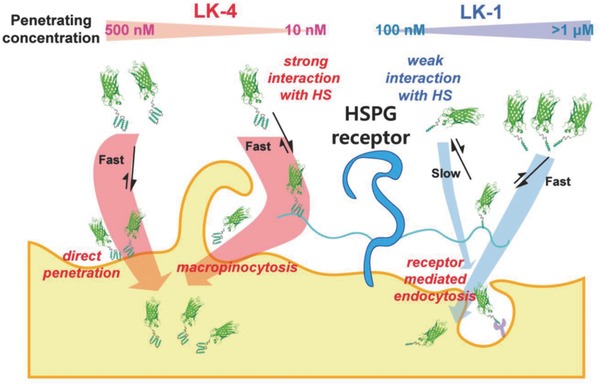
Proposed cell penetrating mechanisms of LK‐1–eGFP and LK‐4–eGFP.

Using the data in the mechanism study, we proposed the internalization mechanisms of LK‐fused proteins (**Figure** **5**). The monomeric LK sequence with cationic charges has affinity with the negatively charged heparan sulfate chains of proteoglycan receptors on the plasma membrane, but this affinity is insufficient enough to induce endocytosis at nanomolar concentrations in a short period. Either a longer time (>6 h) or a higher concentration (>1 × 10^−6^ m) is required to initiate endocytosis. However, the multimerization greatly enhances the affinity with heparan sulfate, and multimeric LK sequences can effectively initiate endocytosis within 0.5–1 h even at concentrations below 50 × 10^−9^ m through the mediation of HSPG although the detailed mediating process is not yet understood. However, the multimeric LK sequences can interact directly with the plasma membrane at concentrations over 500 × 10^−9^ m, still much lower concentrations than the penetrating concentration of Tat ((5–10) × 10^−6^ m), and can induce penetration without ATP consumption.

### 2.3 Control of Adipocyte Differentiation by LK Multimer–Fused Transcription Factor

**Figure 6:**
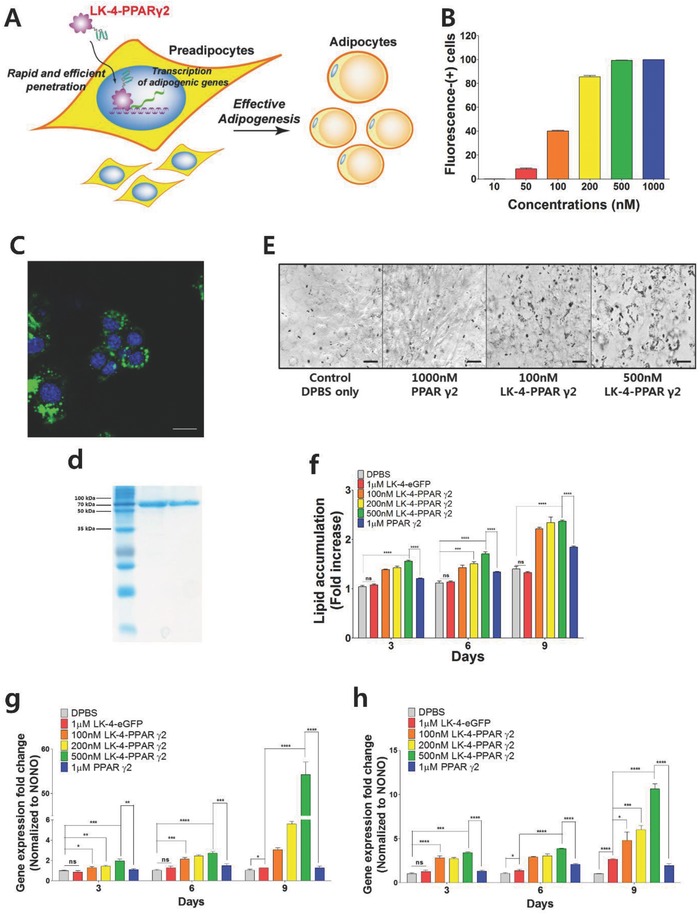
Induction of adipogenic gene expression by LK‐4‐PPAR‐γ  2. A) Schematic representation of induction of adipogenic gene expression by cell permeable LK‐4‐PPAR‐γ   2. B) Cell penetration activities of LK‐4–eGFPs on 3T3‐L1 cells after 12 h of incubation. Fluorescence‐(+) cells were analyzed by FACS. C) A CLSM image of differentiated 3T3‐L1 cells after 12 h of incubation with LK‐4–eGFP at 200 × 10^−9^ m. The nucleus was stained by Hoechst 33342 (blue). The scale bar represents 20 µm. D) The SDS‐PAGE results of purified PPAR‐γ  2 and LK‐4‐PPAR‐γ  2. E) Optical microscopic images of Oil O Red–stained 3T3‐L1 cells treated with PPAR‐γ  2 and LK‐4‐PPAR‐γ  2 at Day 9. DPBS was used for control, and proteins were added to the cells every single day from day 0 to day 8. The scale bar represents 40 µm. F) Quantitative measurement of Oil O Red accumulated in differentiated 3T3‐L1 cells. The Oil O Red was extracted with isopropanol and quantified at 540 nm. Relative mRNA levels of G) adiponectin and H) aP2 genes in differentiated 3T3‐L1 cells treated with PPAR‐γ  2 and LK‐4‐PPAR‐γ  2 every single day from day 0 to day 8. The mRNA expression was quantified by RT‐PCR. Each value was normalized to NONO gene expression. All data points are represented as the average value of three experiments ± standard deviation. (*), (**), (***), and (****) indicate 0.01 ≤ *p* < 0.05, 0.001 ≤ *p* < 0.01, 0.0001 ≤ 2*p* < 0.001, and *p* < 0.0001, respectively. The indication of “ns means that there was no significant difference.

The extraordinary cell penetrating activities of multimeric LK sequences enable bio‐macromolecules to rapidly penetrate cells at nanomolar concentrations. We believe that LK multimer–based delivery could be an effective solution to overcome the barrier of the plasma membrane severely prohibiting the intracellular biomedical effects of bio‐macromolecules at low concentrations. As a proof of concept, we intended to induce adipogenic differentiation though direct transcriptional control using an LK‐4‐fused transcription factor (**Figure** **6**A). The control of mesenchymal stem cell (MSC) differentiation has been of great interest to researchers and clinicians since it has the strong potential to be one of the key techniques for the development of future regenerative medicines.^28^ Among MSC lineages, adipocytes play an essential role in maintaining whole‐body energy homeostasis as part of an endocrine and a paracrine organ although they are recognized as passive participants in the development of obesity.^29^ Adipocyte differentiation involves a series of transcription factors to regulate temporal gene expression. According to the research that has been conducted so far, it is believed that PPAR‐γ  2 acts as the master transcription factor to induce adipocyte differentiation.^30^ However, in most previous studies, adipocyte differentiation was induced either by combinatorial treatment of signaling molecules such as insulin, dexamethasone, and cyclic adenosine monophosphate (cAMP), or gene transfection in order to express the major transcription factors.^31^ As far as we know, direct induction of adipogenesis via the transduction of transcription factors has never been tried. We examined the potency of LK‐4‐fused PPAR‐γ  2 to induce adipogenesis in 3T3‐L1 cells. Preadipocytes are recognized as the representative model in adipocyte differentiation.^32^

First, we estimated the penetration of LK‐4‐fused proteins into 3T3‐L1 cells using LK‐4–eGFP. Although the penetration into 3T3‐L1 cells was somewhat lower than into HeLa cells, more than 80% of cells were fluorescence‐(+) cells at 200 × 10^−9^ m (Figure 6B). The CLSM images also supported the efficient internalization of LK‐4–eGFP into 3T3‐L1 cells at nanomolar concentrations (Figure 6C; Figure S7, Supporting Information). Most of the LK‐4–eGFP was detected in the cytosol, but a small portion was clearly localized in the nucleus, where PPAR‐γ  2 plays a role in adipocyte differentiation.

LK‐4‐PPAR‐γ  2 was prepared by fusing LK‐4 to the N‐terminus of PPAR γ  2 and purified though His‐tag affinity chromatography and gel permeation chromatography (Figure 6D; Figure S8A, Supporting Information). LK‐4‐PPAR‐γ  2 was used to treat 3T3‐L1 cells every single day for adipocyte differentiation, and Dulbecco's phosphate‐buffered saline (DPBS), LK‐4–eGFP, and PPAR‐γ  2 were used as controls. As shown in the optical microscopic images, the fibroblast‐like polar morphology of the 3T3‐L1 cells changed into adipocyte‐like spherical morphology with intracellular lipid droplets after the LK‐4‐PPAR‐γ  2 treatment (Figure 6E; Figure S8B, Supporting Information). Oil Red O staining analysis showed the significant enhancement of lipid accumulation in a concentration‐dependent manner following the treatment of LK‐4‐PPAR‐γ  2 (Figure 6F). At day 9, the treatment of native PPAR‐γ  2 at a high concentration (1 × 10^−6^ m) also showed a marginal increase (1.8‐fold) in lipid accumulation. However, LK‐4‐PPAR‐γ  2 showed a higher lipid accumulation than native PPAR‐γ  2 even at 100 × 10^−9^ m (2.2‐fold). The induction of adipogenesis using LK‐4‐PPAR‐γ  2 was more dramatic when quantified by reverse transcription polymerase chain reaction (RT‐PCR) (Figure 6G,H). We measured the mRNA expression level of two PPAR‐γ  2‐regulated genes, adiponectin and adipocyte protein 2 (aP2), and normalized them to a house‐keeping gene, a non‐pituitary‐specific, octamer transcription factor, neural unc‐86 transcription factor (POU) domain‐containing octamer‐binding protein (NONO) gene.^33^ At day 9, native PPAR‐γ  2 showed only a 2‐fold enhancement of adiponectin and aP2 expression at 1 × 10^−6^ m, whereas LK‐4‐PPAR‐γ  2 showed 3‐, 6‐, and 55‐fold enhancements of adiponectin and 3‐, 6‐, and 11‐fold enhancements of aP2 at the concentrations of 100 × 10^−9^, 200 × 10^−9^, and 500 × 10^−9^ m, respectively. The results strongly supported that LK‐4‐PPAR‐γ is internalized into 3T3‐L1 cells and can initiate the transcription of adipocyte‐specific genes for differentiation into adipocyte cells.

The potential of our multimeric LK‐4‐mediated protein transduction for the adipocyte differentiation was compared with the conventional combinatorial treatment of insulin, dexamethasone, and isobutylmethylxanthin (IBMX) (Figure S9, Supporting Information). The induction of lipid accumulation by LK‐4‐PPAR‐γ  2 at 200 × 10^−9^ m was slightly lower than the conventional method. However, the co‐treatment of LK‐4‐PPAR‐γ  2 and sodium butyrate, a well‐known histone deacetylase inhibitor to enhance transcription efficiency, could boost the lipid accumulation up to the level similar to the insulin–dexamethasone–IBMX method. We expected that more effective induction of adipogenic genes could be accomplished by future combinatorial treatment of transcription factors: for example, PPAR‐γ, Ccaat‐enhancer‐binding protein (C/EPB), and Krueppel‐like factor 5 (Klf5).^34^

## 3 Conclusion

We constructed a series of CPPs based on fusing multimeric α‐helical amphiphatic LK sequences with cargo proteins. The fusion proteins can penetrate the cell membrane within 30 min at 100–1000‐fold lower concentrations than the penetrating concentrations of Tat‐fused proteins. The multimeric CPP‐fused proteins were internalized by ATP‐dependent macropinocytosis through strong interactions with HSPG receptors at low nanomolar concentrations and through ATP‐independent direct penetration at high nanomolar concentrations. Owing to the outstanding penetrating activities of the multimeric CPPs, we successfully delivered a transcription factor (PPAR‐γ  2) in an active form and induced adipogenesis at nanomolar concentrations. The discovery of multimeric CPPs may be a major breakthrough and could facilitate scientific studies on controlling intracellular biological reactions and the biomedical applications of delivering difficult therapeutic reagents into intracellular targets though rapid and efficient penetration at 2–3 orders of magnitude lower concentrations than standard CPPs.

## 4 Experimental Section

*Cell Lines and Cell Culture*: HeLa (human cervix epithelial carcinoma), HEK 293T (human embryotic kidney), MDA‐MB‐231 (human breast cancer) cells were purchased from the American Type Culture Collection (ATCC) and maintained in high glucose Dulbecco's modified Eagle's medium (DMEM) containing 10% (v/v) fetal bovine serum (FBS) (Wellgene) at 37 °C in the presence of 5% CO_2_. CHO K1 cells were purchased from Korean Cell Line Bank (KCLB) and cultured in Ham's F12 medium containing 10% FBS at 37 °C in the presence of 5% CO_2_. HEK 293s GnTI^−^ (human embryotic kidney) cells (ATCC) were cultured under standard condition in Dulbecco's modified Eagle's medium and F12 ratio 1:1 medium containing 10% FBS at 37 °C in the presence of 5% CO_2_. 3T3‐L1 (*mus musculus* embryo fibroblast) (KCLB) cells were maintained in Dulbecco's modified Eagle's medium containing 10% bovine calf serum (BCS) (Wellgene) and incubated at 37 °C in the presence of 5% CO_2_.

*Peptide Synthesis—Solid Phase Peptide Synthesis*: For fluorenylmethyloxycarbonyl protecting group (Fmoc) deprotection, the resins were placed in a microwave vessel and irradiated for 2 min (ramping time for 1 min) at 5 W power. For the coupling step, the resins were microwave‐irradiated for 5 min (ramping time for 2 min) at 5 W power. The temperature was set at 35 °C for both steps.

*Peptide Synthesis—Dimerization of Peptides*: Dimeric bundle peptides were prepared by air oxidation as previously described.[[qv: 15a,35]] Briefly, cysteine‐containing peptide monomer was dissolved in 0.1 m deaerated ammonium bicarbonate to give a final concentration of 1 mg mL^−1^, and the mixture was incubated to stand open to atmosphere until the reaction was complete. Parallel and antiparallel dimers were obtained and shown to be separated by HPLC using a C18 column (Zorbax C18, 3.5 mm, 4.6 × 150 mm) as the stationary phase and buffer A (water with 0.1%, v/v trifluoroacetic acid (TFA)) and buffer B (acetonitrile with 0.1%, v/v TFA) as the mobile phase. Parallel dimers were major products (antiparallel dimers were obtained less than 5% judged by HPLC traces) and found to be relatively nonpolar than antiparallel minor dimers as described previously.^22^ Dimeric peptides were confirmed by using matrix‐assisted laser desorption/ionization‐time of flight (MALDI‐TOF) and purified by a preparative HPLC.

*Peptide Synthesis—Fluorescence‐Labeled Peptides*: The dye 5‐carboxytetramethylrhodamine (5‐TAMRA) (Merck Millipore) was used to lead fluorescently labeled peptides. The fluorescent dye was coupled at the N‐terminus of each peptide using 2‐(6‐choloro‐1‐*H*‐benzotriazole‐1‐yl)‐1,1,3,3‐tetramethylaminum hexafluorophosphate (HCTU) activation. Briefly, 5‐TAMRA (2 equiv. relative amount to Fmoc‐deprotected N‐terminus amine) was dissolved in anhydrous dimethylformamide (DMF) to a final concentration of 0.1–0.5 m and activated with HCTU (2 equiv.), 1‐hydroxybenzotriazole (HOBt, 2 equiv.), and diisopropylethylamine (4 equiv.). The activated 5‐TAMRA solution was added to the Fmoc‐deprotected resin and stirred for 2 h at room temperature. When the reaction was complete, peptides were cleaved from resins followed by the normal procedure. For dimer peptides, 5‐TAMRA was labeled on only one strand (Figure S10, Supporting Information).

*Construction of the Expression Vectors*: A bacterial expression of vectors containing hexahistidine leader sequence was designed. A gene encoding eGFP in the C2–eGFP vector was amplified by PCR using the C2–eGFP as a template and oligonucleotide primers containing *BamHI* and *XhoI* (New England Biolabs) restriction sites. The pET28b vector and the amplified eGFP gene were digested with *BamHI* and *XhoI* and ligated (New England Biolabs) to make pET28b–eGFP as a negative control. Tat‐encoded gene (Cosmo Genetech) was amplified by PCR using primers containing *NdeI* and *BamHI*. Both the vector and PCR fragment were digested by *NdeI* and *BamHI* restriction enzymes and ligated to make pET28b–Tat–eGFP vector as a positive control. For LK‐1–eGFP, a gene encoded LK‐1 in the produced at the University of California (pUC) vector was produced by gene synthesis (Cosmo Genetech). The LK‐1 gene was amplified using PCR and cloned into a pET28 vector, and the gene encoding eGFP was inserted later. For LK‐2–eGFP, a gene encoded LK‐2 in the pUC vector was produced by gene synthesis, cloned into pET28b–eGFP. For LK‐3–eGFP, the PCR product of LK‐2 gene was inserted into pET28b‐LK‐1–eGFP. For LK‐4–eGFP, the PCR product of LK‐2 gene was ligated with pET28b‐LK‐2–eGFP. For LK‐5–eGFP and LK‐6–eGFP, the LK‐4 fragment without restriction sites was synthesized by gene synthesis. Later, the LK‐4 sequence was inserted into LK‐1–eGFP and LK‐2–eGFP to produce LK‐5–eGFP and LK‐6–eGFP, respectively. For PPAR‐γ  2 vectors, pBABE puro PPAR‐γ  2 vector was purchased from addgene. pET28b vector was digested by *NdeI* and *XhoI* and ligated with the PPAR‐γ  2 PCR fragment to construct pET28b‐PPAR‐γ  2 vector. pET28b–LK‐4–PPAR‐γ  2 vector was generated by ligation between the PCR products of pET28b–LK‐4–eGFP and PPAR‐γ  2 with *SacI* and *XhoI* primers. Each PCR primer sequence was summarized in Table S2 of the Supporting Information.

*Purification of Recombinant Proteins*: All recombination proteins were expressed using bacterial systems. *E. coli Rosetta* (DE3) pLysS (Novagen) were transformed with plasmids encoding recombinant proteins and incubated in 10 mL of luria‐bertani (LB) broth containing kanamycin and chloramphenicol for 16 h at 37 °C. Then, the cultures were transferred into 1 L of fresh LB medium and incubated at 37 °C for further 2–3 h until OD_600_ reached 0.4–0.6. Isopropyl‐β‐thiogalactopyranoside (1 × 10^−3^ m) was added to the media. The cells were incubated overnight at 16 °C with shaking at 190 rpm, subsequently. The cells were then harvested by centrifugation at 6000 rpm for 10 min at 4 °C and re‐suspended in a lysis buffer (20 × 10^−3^ m Tris, 500 × 10^−3^ m NaCl, 35 × 10^−3^ m imidazole, 0.05% Triton X‐100, pH 7.0). After the suspension was sonicated by the ultrasonic processor (Sonic & Materials, Inc.), the supernatant was obtained by centrifugation at 13 000 rpm for 20 min at 4 °C. The supernatant was filtered through 0.22 µm syringe filters, and the 6‐His‐tagged proteins were purified via nichel‐nitrilotriacetic acid (Ni‐NTA) affinity chromatography (GE Healthcare) and desalted with a solution containing 20 × 10^−3^ m Tris, 200 × 10^−3^ m KCl, 1 × 10^−3^ m dithiothreitol (DTT) and 10% glycerol through a 30K Amicon centrifuge filter (Milipore). The protein solution could be further purified by a Superdex 200 16/60 (GE Healthcare) size column with an eluent containing 20 × 10^−3^ m Tris and 400 × 10^−3^ m NaCl (pH 7.5). Protein concentrations were measured using the Quick Bradford assay (Bio‐Rad). Purified proteins were stored in a solution containing 20 × 10^−3^ m Tris, 200 × 10^−3^ m KCl, 1 × 10^−3^ m DTT, and 10% glycerol.

*Measurement of Cell Penetration Activities*: Cells (8 × 10^4^ cells per well) were seeded in 24‐well plates and incubated in DMEM containing 10% FBS overnight. Recombinant eGFP proteins were added to the cells at various concentrations and further incubated for 12 h. For the experiment of penetrating kinetics, the incubation time was varied. For the endocytosis inhibition assay, cells were pretreated with endocytosis‐inhibiting conditions before the protein treatment: treatment of 10 × 10^−3^ m sodium azide (NaN_3_), 50 × 10^−6^ m amiloride, or 100 × 10^−6^ m wortmannin for 3 h. For the inhibition assay at low temperatures, cells were preincubated at 4 °C for 3 h, then LK fusion proteins were treated for 1 h at 4 °C. After the protein treatment, the cells were washed thoroughly with DPBS (×3) and incubated with trypsin–ethylenediaminetetraacetic acid (EDTA) (0.25%) for 10 min at 37 °C to digest the proteins bound to the cell surface. Detached cells were harvested and centrifuged at 13 000 rpm for 10 min and suspended in DPBS containing 2% FBS. The FACS analysis was performed on FACS Calibur (Becton Dickinson, USA). A total of 1 × 10^4^ cells were assessed for each sample and dead cells were excluded from the analysis.

*Confocal Laser Fluorescence Microscopy*: Cells (1.5 × 10^4^ cells per well) were seeded on confocal dish (SPL) and incubated at 37 °C, 5% CO_2_. After 24 h, the cells were treated with recombinant eGFP proteins in fresh complete medium at 37 °C at various concentrations. A Hoechst 33342 dye solution (Thermo Fisher Scientific) was added to the cells at a final concentration of 1 µg mL^−1^, and the cells were incubated for 10 min. The cells were washed thoroughly with DPBS (×3) to remove proteins bound to the cell surface. After the addition of fresh complete media, images were acquired using an LSM 510 laser scanning confocal microscope (Carl Zeiss, Oberkochen, Germany) with a 400× objective.

*LDH Assay*: HeLa cells (1 × 10^4^ cells per well) were seeded in 96‐well plates and incubated for 24 h. The media was exchanged and cells were treated with proteins for further 24 h. The LDH assay was performed with the Pierce LDH Cytotoxicity Assay Kit (Thermo Scientific) following manufacturer's protocols. The absorbance was measured using the multifunctional microplate reader Infinite 200PRO (TECAN).

*Heparin Interaction Assay*: Heparin interaction assay was performed with the biolayer interferometer, the BLItz System (Pall ForteBio). The surface of AR2G tip (Octet biosensors) was activated in deionized water for 30 s and then exposed to a solution of branched polyethylenimine (1 × 10^−3^ m in phosphate‐buffered saline (PBS), *M*_w_ = 25 000, Sigma‐Aldrich) for 250 s for the formation of positively charged surface. Then, a heparin sodium salt solution (100 × 10^−6^ m in PBS, Millipore 375095) was allowed to flow on the surface for another 250 s for the immobilization of heparin through electrostatic interaction. The multilayered surface was washed with PBS for 60 s. A protein sample was associated with the sensor for 100 s by the flow of protein solutions (2.5 × 10^−6^ m in PBS) and dissociated for 100 s by washing the surface with PBS.

*XylT‐I Knockdown Assay*: HeLa cells (5 × 10^4^ cells per well) were seeded in 24‐well plates and incubated for 24 h. The cells were transfected with XylT‐I siRNA (40 pmol, Santa Cruz) using Lipofectamine 2000 (1 µL, Invitrogen) following the manufacturer's protocols. After the treatment of the XylT‐I siRNA complex for 48 h, LK‐4–eGFP protein was treated for 1 h at various concentrations. Control siRNA (Santa Cruz) was used as a control. The knockdown assay in HEK293T and HEK293s GnTi^−^ cells was performed in a similar manner except for the initial cell density (1.5 × 10^4^ cells per well).

*Cell Viability Assay*: A cytotoxicity assay was performed using the cell counting kit‐8 (Dojindo, Korea). HeLa cells (5 × 10^3^ cells per well) were seeded in 96‐well plates and incubated for 24 h. Then, growth media were replaced with fresh culture media (100 µL per well) containing 1% FBS, and the cells were treated with samples with various concentrations of proteins for 24, 48, and 72 h. Following the incubation, 10 µL of CCK‐8 solution was added, and the cells were incubated at 37 °C for 1 h. The absorbance at 450 nm was measured using a microplate reader (Molecular Device Co., Menlo Park, CA).

*Adipocyte Differentiation*: 3T3‐L1 cells (2 × 10^4^ cells per well) were grown in high glucose DMEM supplemented 10% FBS in a 48‐well plate. At confluence (day 0), the cells were first treated with recombinant proteins. Protein was treated every single day from day 0 to day 8. The cells were washed once in DPBS (Wellgene) and fixed with 4% paraformaldehyde in DPBS for 20 min, followed by the Oil O Red staining method described previously.^36^ Differential interference constrast (DIC) images were obtained using an inverted microscope (Zeiss Axio Observer Z1). For RT‐PCR, total RNA was isolated from the cells using a NucleoSpin RNA kit (MN). 1 µg of RNA was reverse transcribed using the QuantiTech reverse transcription kit (Quiagen). SYBR green reactions using KAPA SYBR fast qPCR kit (Biosystems) were performed with BioRad CFX connect real‐time PCR equipment (BioRad). Relative expression of mRNA was determined after normalization to total RNA amount. The primers were summarized in Table S3 of the Supporting Information.

*Statistical Analysis*: The data were analyzed using two‐tailed Student's *t*‐tests; *p*‐values of <0.05 were considered as significant differences. Differences are presented on graphs as *, **, ***, and ****, which indicate 0.01 ≤ *p* < 0.05, 0.001 ≤ *p* < 0.01, 0.0001 ≤ *p* < 0.001, and *p* < 0.0001, respectively. The indication of “ns” means that there was no significant difference.

## Conflict of Interest

The authors declare no conflict of interest.

## Supporting information

Supplementary
