# Supplementary material for: Multimeric Amphipathic α‐Helical Sequences for Rapid and Efficient Intracellular Protein Transport at Nanomolar Concentrations
Source: Adv Sci (Weinh). 2018 Jun 19;5(8):1800240. doi: 10.1002/advs.201800240 (PMC6096998; doi:10.1002/advs.201800240)
Supplement: Supplementary file 1 — Supplementary [file ADVS-5-1800240-s001.pdf]

Copyright WILEY-VCH Verlag GmbH & Co. KGaA, 69469 Weinheim, Germany, 2018.

ADVANCED SCIENCE
Open Access

Supporting Information

for *Adv. Sci.,* DOI: 10.1002/advs.201800240

Multimeric Amphipathic α-Helical Sequences for Rapid and Efficient Intracellular Protein Transport at Nanomolar Concentrations

Jae Hoon Oh, Seung-Eun Chong, Sohee Nam, Soonsil Hyun, Sejong Choi, Hyojun Gye, Sangmok Jang, Joomyung Jang, Sung Won Hwang, Jaehoon Yu, and Yan Lee**

Supporting Information (SI)

Multimeric Amphipathic α-Helical Sequences for Rapid and Efficient Intracellular Protein Transport at Nanomolar Concentrations

By Jae Hoon Oh, Seung Eun Chong, Sohee Nam, Soonsil Hyun, Sejong Choi, Hyojun Gye, Sangmok Jang, Joomyung Jang, Sung Won Hwang, Jaehoon Yu, * Yan Lee*

(A)

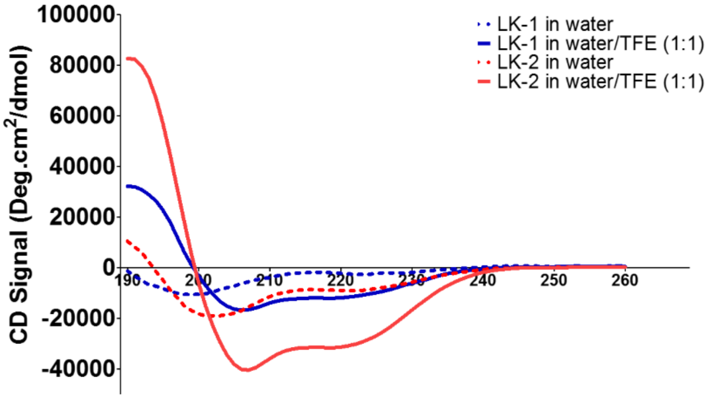

(B)

	Ellipticity (mdeg) (raw data)	Ellipticity at 222nm	α–Helicity Contents (%)
**LK-1 in water**	-4.2	-2600	**14**
**LK-1 in 50% water/TFE**	-18	-11000	**37**
**LK-2 in water**	-29	-9200	**31**
**LK-2 in 50% water/TFE**	-97	-30000	**85**

Fig. S1. Circular Dichroism (CD) of LK-1 and LK-2 A) CD spectra of LK-1 and LK-2 peptides in water and in 50% trifluoroethanol(TFE)/water mixture. B) Calculated α-helicities of LK-1 and LK-2. The CD was measured at the peptide concentration of 100 μM.

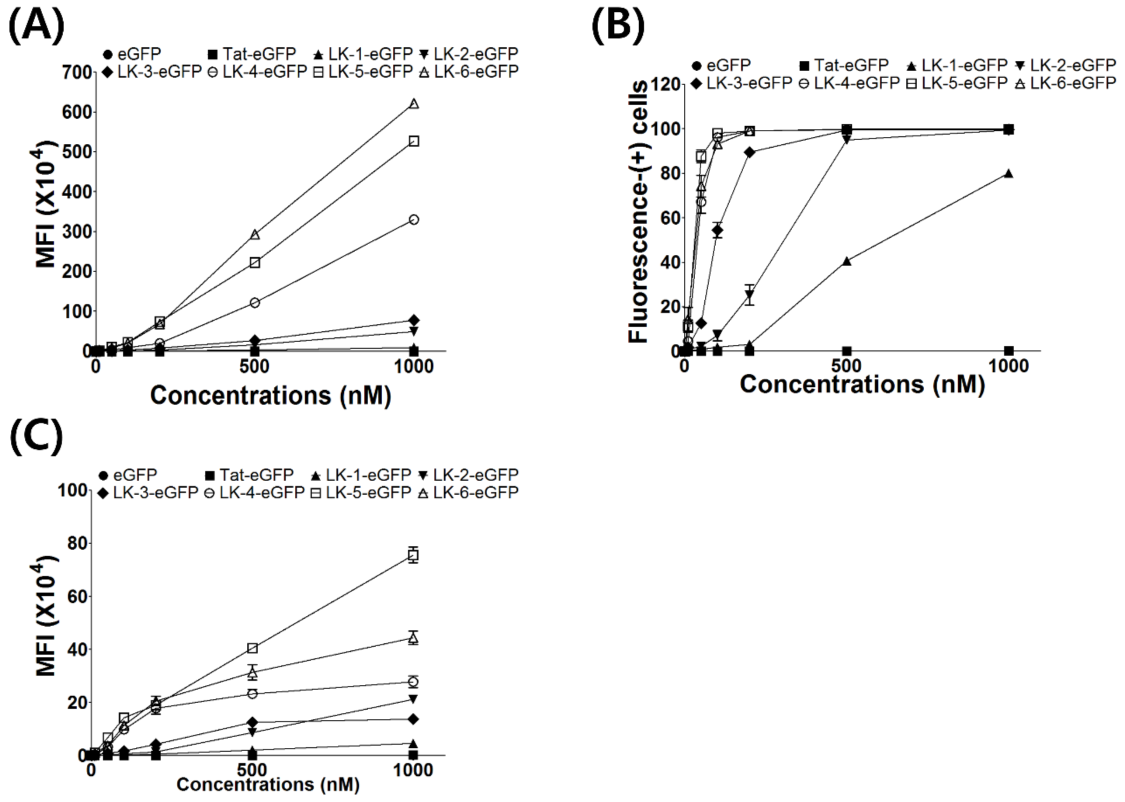

Fig. S2. Cell penetration activities of CPP-fused eGFPs on HeLa cells and HEK293T cells after 12 h-incubation. A) HeLa cells were incubated with CPP-fused eGFPs at various concentrations (10–1000 nM) for 12 h. FACS data were represented as mean fluorescence intensity (MFI). All data points are represented as the average value of three experiments ± standard deviation. B) HEK293T cells were incubated with CPP-fused eGFPs at various concentrations (10–1000 nM) for 12 h. FACS data were represented as fluorescence-(+) cell percentages and C) mean fluorescence intensity (MFI). All data points are represented as the average value of three experiments ± standard deviation.

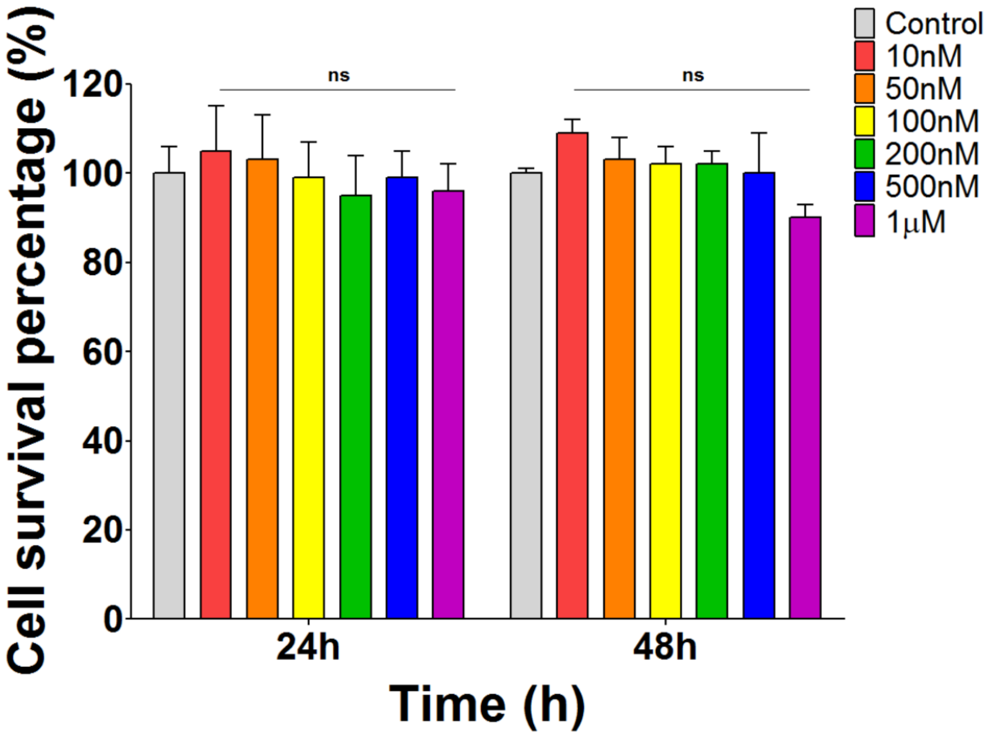

Fig. S3. Relative viability of HeLa cells treated with LK-4-eGFP. HeLa cells were incubated with LK-4-eGFP at various concentrations for 24 h and 48 h. All data points are represented as the average value of three experiments ± standard deviation. The indication of 'ns' means that there was no significant difference with the control.

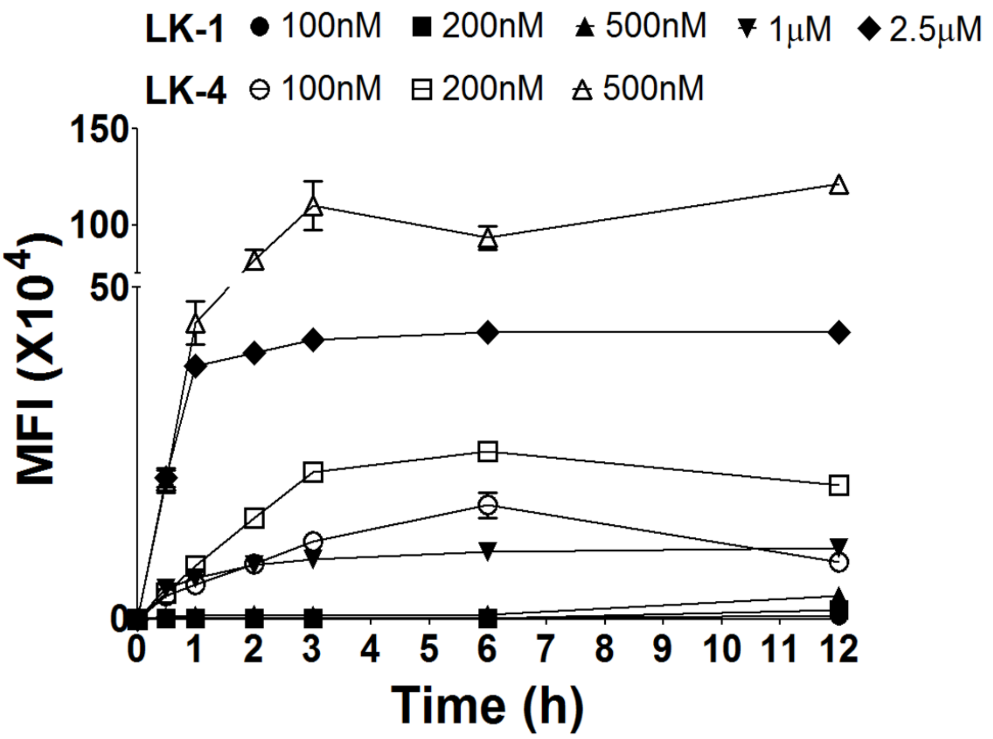

Fig. S4. Cell penetrating kinetics of LK-1-eGFP and LK-4-eGFP on HeLa cells at various concentrations. FACS data were represented as mean fluorescence intensity (MFI). All data points are represented as the average value of three experiments ± standard deviation.

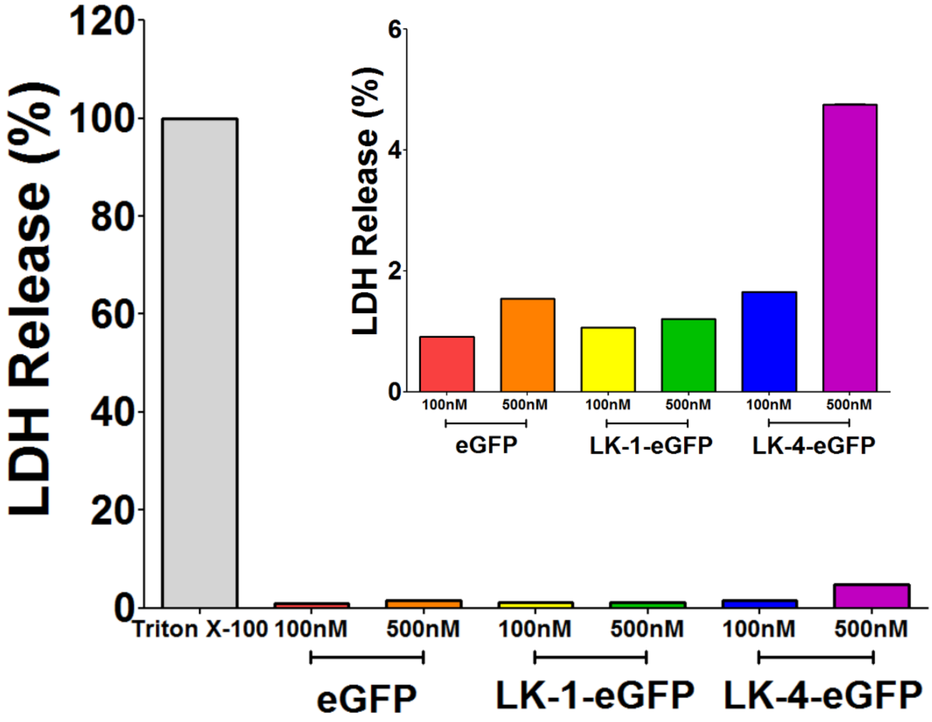

Fig. S5. Membrane destabilization by LK-eGFPs. The LDH assay results on HeLa cells treated with LK-1-eGFP and LK-4 eGFP for 24 h. All data points are represented as the average value of three experiments ± standard deviation.

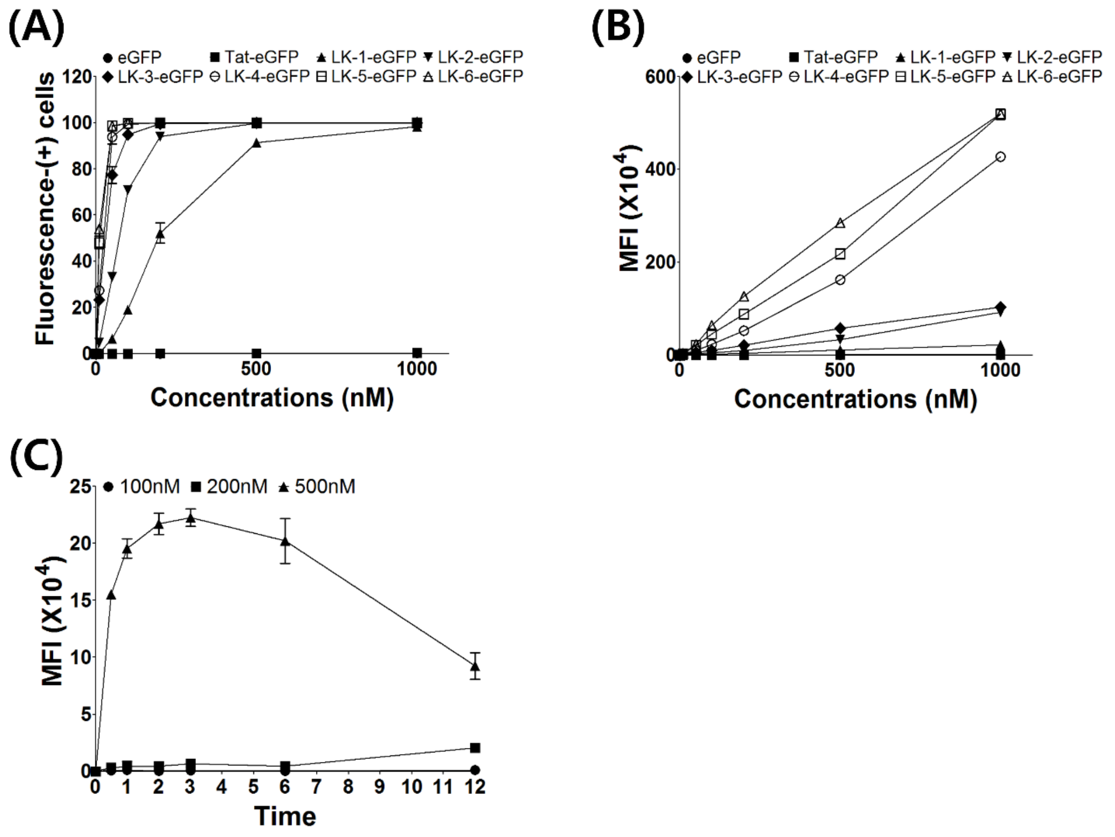

Fig. S6. Cell penetration activities of CPP-fused eGFPs on MDA-MB-231 cells and CHO K1 cells after 12 h-incubation. MDA-MB-231 cells were incubated with CPP-fused eGFPs at various concentrations (10–1000 nM) for 12 h. FACS data were represented as A) fluorescence-(+) cell percentages and B) mean fluorescence intensity (MFI). C) Cell penetrating kinetics of LK-4-eGFP on CHO-K1 cells at various concentrations. FACS data were represented as mean fluorescence intensity (MFI). All data points are represented as the average value of three experiments ± standard deviation.

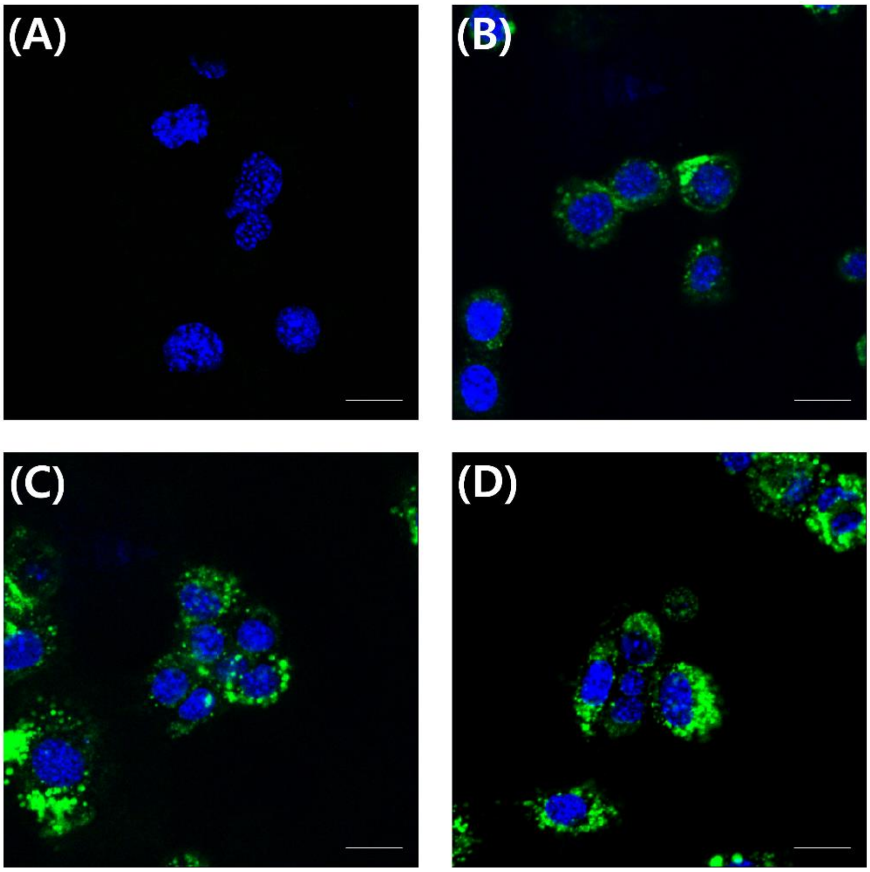

Fig. S7. CLSM images of differentiated 3T3-L1 cells after 12 h-incubation with CPP-fused-eGFP. CLSM images of differentiated 3T3-L1 cells after 12 h-incubation with A) Tat-eGFP at 1 μM and LK-4-eGFP at B) 100 nM, C) 200 nM and D) 500 nM. The nucleus was stained by Hoechst 33342 (blue). The scale bar represents 20 μm

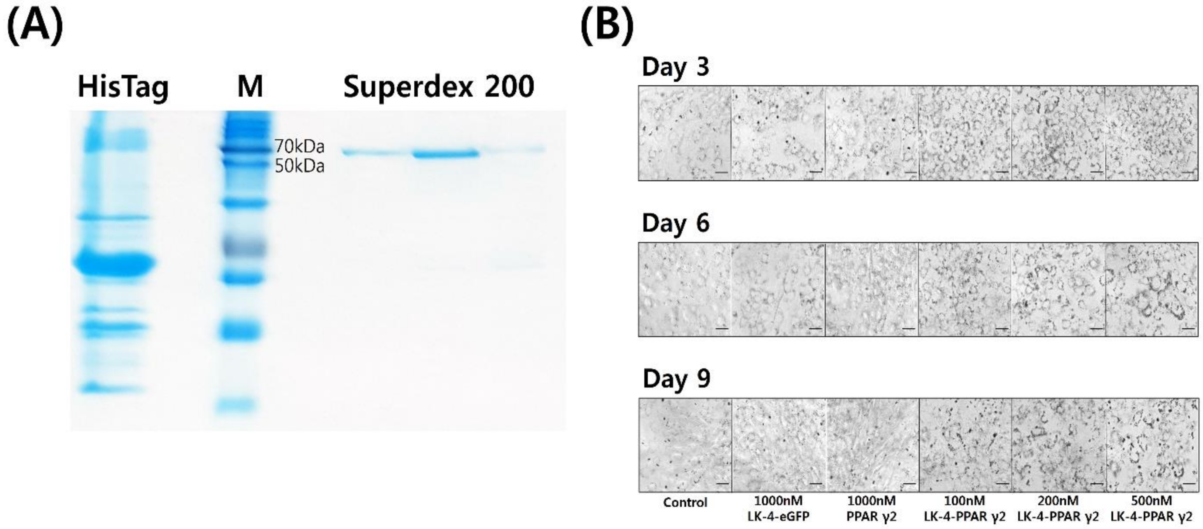

Fig. S8. Purification of LK-4-PPAR-γ2 and Optical microscopic images of Oil O Red-stained 3T3-L1 cells. A) Proteins were initially purified from with a Ni-NTA column (left). Next, the protein mixture was further purified with a Superdex200 size column (right). B) DPBS was used for control and proteins were added to the cells every single day from Day 0 to Day 8. The scale bar represents 40 μm.

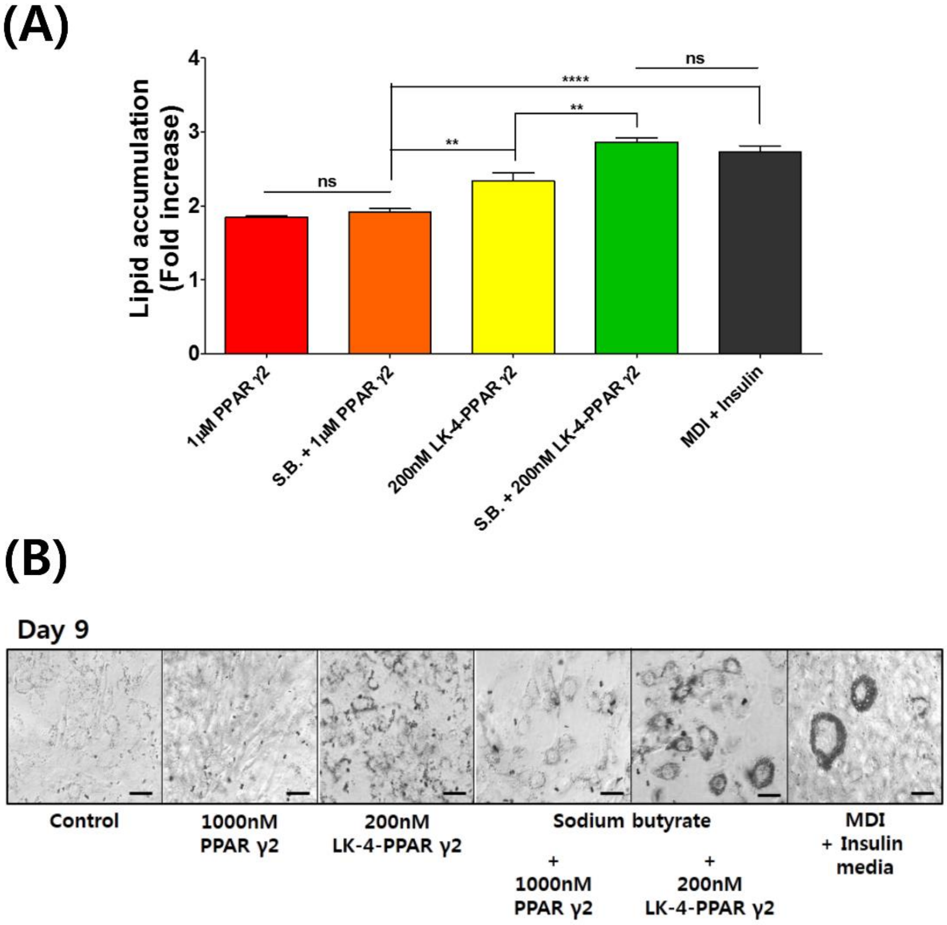

Fig. S9. Comparison of adipocyte differentiation efficiency with the MDI method. A) Quantitative measurement of Oil O Red accumulated in differentiated 3T3-L1 cells at Day 9. The Oil O Red was extracted with isopropanol and quantified at 540 nm. All data points are represented as the average value of three experiments ± standard deviation. (**) and (****) indicate $0.001 \leq p < 0.01$ and $p < 0.0001$, respectively. The indication of 'ns' means that there was no significant difference. B) Optical microscopic images of Oil O Red-stained 3T3-L1 cells at Day 9. The scale bar represents 40 μm. S.B. and MDI represent sodium butyrate and methylisobutyxanthine/dexamethasone induction, respectively.

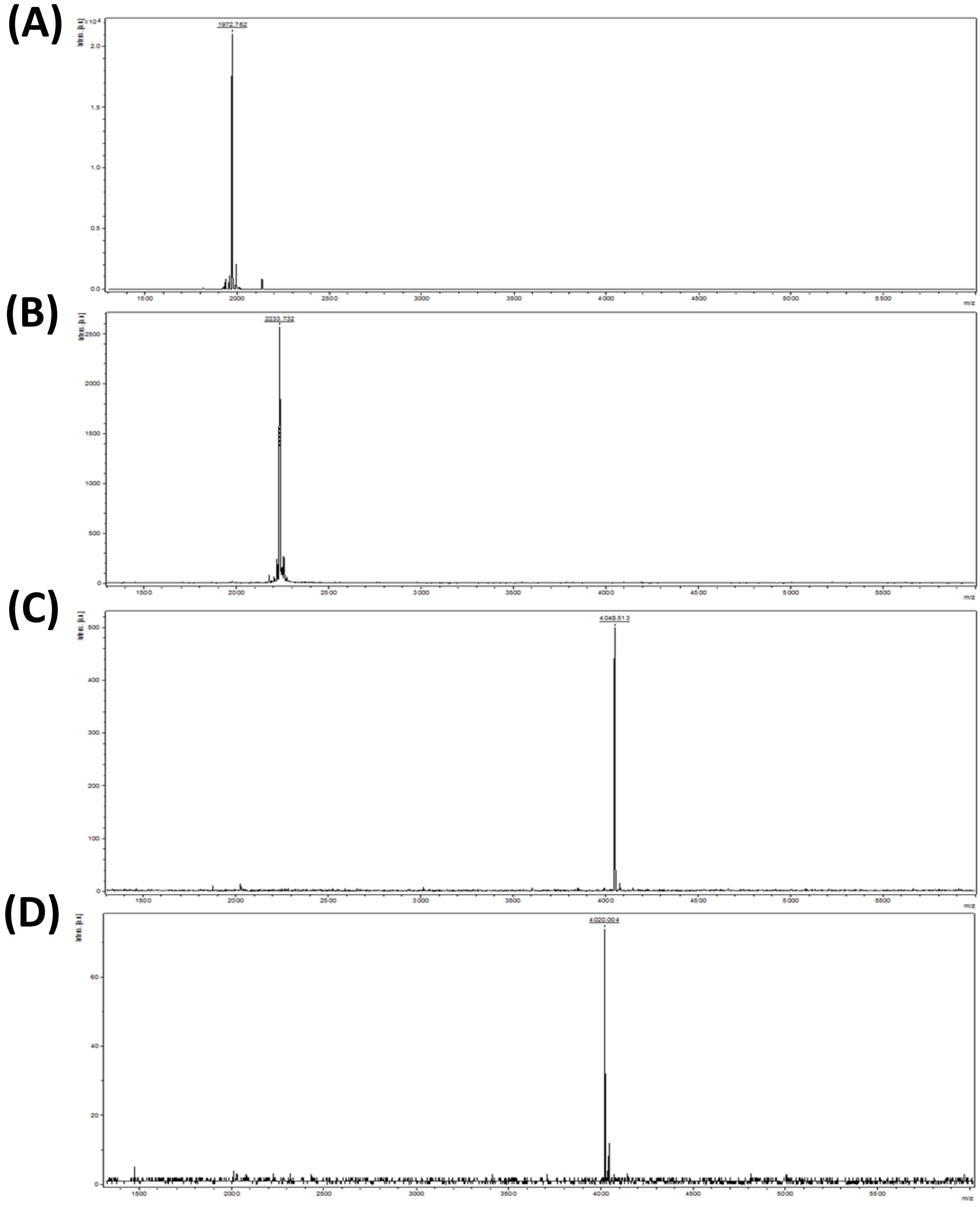

Fig. S10. MALDI-TOF MS spectra of synthesized peptides. A) TAMRA-Tat. MS $[M+H]^+$: 1970.9 (calcd.), 1972.8 (found). B) TAMRA-LK-1. MS $[M+H]^+$: 2232.5 (calcd.), 2233.7 (found). C) TAMRA-LK dimer. MS $[M+H]^+$: 4049.5 (calcd.), 4048.5 (found). D) TAMRA-LK-2. MS $[M+H]^+$: 4020.9 (calcd.), 4020.0 (found)

LK Multimers	Amino acid sequences (Linker)
LK-3 (trimer)	LKKLLKLLKKLLKLGGLKKLLKLLKKLLKLAGGSEFLKKLLKLLKKLLKLAG
LK-4 (tetramer)	LKKLLKLLKKLLKLGGLKKLLKLLKKLLKLAGGSEFLKKLLKLLKKLLKLGGLKKLLKLLKKLLKLAG
LK-5 (pentamer)	LKKLLKLLKKLLKLGGLKKLLKLLKKLLKLAGGSEFLKKLLKLLKKLLKLGGLKKLLKLLKKLLKLAGKSSDPNSSSLKKLLKLLKKLLKLAG
LK-6 (hexamer)	LKKLLKLLKKLLKLGGLKKLLKLLKKLLKLAGGSEFLKKLLKLLKKLLKLGGLKKLLKLLKKLLKLAGKSSDPNSSSLKKLLKLLKKLLKLGGLKKLLKLLKKLLKLAG

Table S1. Amino acid sequences of multimeric LK peptides.

Gene constructs	Amplified gene	Primers (5′ to 3′)	Restriction Enzymes
pET28-eGFP	eGFP	For: CGCGGATCCGTGAGCAAGGGCGAG	BamHI
		Rev: GAGCTGTACAAGTAACTCGAGCAC	XhoI
pET28-Tat-eGFP	Tat	For: AGCCATATGTACGGCC	NdeI
		Rev: CCGTCGCGGATCCGTG	BamHI
pET28-LK-1-eGFP	LK-1	For: TCCGAATTCACTGAAGAAACT	EcoRI
		Rev: AACTGGCTGGTGGATCCGTG	BamHI
pET28-LK-2-eGFP	LK-2	For: AGCCATATGCTCAAGAAACTGC	NdeI
		Rev: GAAACTGGCTGGTGGATCCGTG	BamHI
pET28-LK-3-eGFP	LK-3	For: TCCGAATTCACTGAAGAAACT	EcoRI
		Rev: GAGCTGTACAAGTAACTCGAGCAC	XhoI
pET28-LK-4-eGFP	LK-4	For: TCCGAATTCCTCAAGAAACTGCTGAAG	EcoRI
		Rev: GAGCTGTACAAGTAACTCGAGCAC	XhoI
pET28-LK-5-eGFP	LK-5	For: TTCGAGCTCACTGAAGAAACTGCTCA	SacI
		Rev: GAGCTGTACAAGTAACTCGAGCAC	XhoI
pET28-LK-6-eGFP	LK-6	For: TTCGAGCTCACTCAAGAAACTGCTGA	SacI
		Rev: GAGCTGTACAAGTAACTCGAGCAC	XhoI
pET28-PPAR γ2	PPAR γ2	For: AGCCATATGATGGGTGAAACTCTGG	NdeI
		Rev:GTGCTCGAGTTAATACAAGTCCTTGTAGATCTCC	XhoI
pET28-LK-4 -PPAR γ2	LK-4	For: TAACTCGAGCACCACC	XhoI
		Rev:CATGAGCTCGGATCCACCAGCCAGTTTCAAC	SacI
pET28-LK-4 -PPAR γ2	PPAR γ2	For: TCCGAGCTCATGGGTGAAACTCTGGG	SacI
		Rev: GTGCTCGAGTTAATACAAGTCCTTGTAGATCTCC	XhoI

Table S2. Primer sequences for the recombinant DNA constructs.

RT qPCR target gene	Primers (5′ to 3′)
NONO (house keeping gene)	For: UGCUCCUGUGCCACCUGGUACUC
	Rev: CCGGAGCUGGACGGUUGAAUGC
aP_2	For: UGCCACAAGGAAAGUGGCAG
	Rev: CUUCACCUUCCUGUCGUCUG
Adiponectin	For: AAGAAGGACAAGGCCGUUCUCUU
	Rev: GCUAUGGGUAGUUGCAGUCAGUU

Table S3. Primer sequences for RT-qPCR.
